# Microstructure and Properties of PZT Films with Different PbO Content—Ionic Mechanism of Built-In Fields Formation

**DOI:** 10.3390/ma12182926

**Published:** 2019-09-10

**Authors:** Nikolay Mukhin, Dmitry Chigirev, Liubov Bakhchova, Andrey Tumarkin

**Affiliations:** 1 Institute for Micro and Sensor Systems, Otto-von-Guericke University, 39106 Magdeburg, Germany; 2 Faculty of Electronics, Saint Petersburg Electrotechnical University “LETI”, Saint Petersburg 197376, Russia; 3 Institute for Automation Engineering, Otto-von-Guericke University, 39106 Magdeburg, Germany

**Keywords:** PZT, lead oxide, heat treatment, grain boundaries, self-polarization

## Abstract

Experimental studies were conducted on the effects of lead oxide on the microstructure and the ferroelectric properties of lead zirconate-titanate (PZT) films obtained by the method of radio frequency (RF) magnetron sputtering of a ceramic PZT target and PbO_2_ powder with subsequent heat treatment. It is shown that the change in ferroelectric properties of polycrystalline PZT films is attributable to their heterophase structure with impurities of lead oxide. It is also shown that, even in the original stoichiometric PZT film, under certain conditions (temperature above 580 °C, duration greater than 70 min), impurities of lead oxide may be formed. The presence of a sublayer of lead oxide leads to a denser formation of crystallization centers of the perovskite phase, resulting in a reduction of the grain size as well as the emergence of a charge on the lower interface. The formation of the perovskite structure under high-temperature annealing is accompanied by the diffusion of lead into the surface of the film. Also shown is the effect of the lead ions segregation on the formation of the self-polarized state of thin PZT films.

## 1. Introduction

Ferroelectric materials in both bulk and thin-film forms are widely used in electronics and technology. They are used in non-volatile memory, dynamic random-access memory, capacitors, devices designed for volume and surface acoustic waves, deflectors, infrared radiation detectors, optical fibers and delay lines, frequency multipliers, and in photovoltaic and other devices [1,2,3,4]. At present, work aimed at obtaining and studying the properties of ferroelectric materials is being conducted in many countries in major state scientific centers [Sandia Lab (US), National Renewable Energy Lab (US), National Aeronautics and Space Administration (NASA) (US)] and with the participation of commercial companies [Intel (US), Toshiba (Japan), Gennum Inc. (Canada), Paratek Inc. (US)].

Solid solutions of lead zirconate-titanate [Pb(Zr,Ti)O_3_, abbreviated as PZT] are among the most widely available ferroelectric materials. Some oxides, e.g., PbO, TiO_2_, ZrO_2_, that are part of PZT have unique electrophysical and mechanical characteristics and a nanoscale structure in the case of the formation of thin layers [5,6,7,8]. PZT is interesting because it allows almost all applications of ferroelectrics to be covered due to the fact that, depending on the composition [9] and the alloying additives [10,11], it is possible to synthesize PZT with varied physical properties and material parameters over a very wide range while adapting them to the requirements of the specific practical application. Such properties are associated with high dielectric permeability and residual polarization, a large number of transition cycles, high breakdown voltage, pyroelectric and piezoelectric effects, electro-optical phenomena, photoelectricity, etc.

Because of the wide variety of processes involved in the formation of the microstructure and the properties of PZT, various research groups still continue to investigate the PZT material [11,12,13,14,15].

The variety of studies to control the properties and the structure of PZT based films can be divided into several branches. The first is to control the properties and the structure of PZT films by manipulating their stoichiometry and impurities [10,11]. The second is the production of epitaxial films [16]. The third is the study of interfaces and particularly the influence of mechanical stresses at the boundary of PZT films with the substrate and the sublayers [17,18]. Fourth is a study of size effects [19]. Fifth is the creation of ferroelectric composites [20], metamaterials, and superlattices such as in [21,22,23], where PZT can also be used. The latter branches are a field for obtaining new effects and new devices.

In this paper, we focused only on the study of the effect of non-stoichiometry on the properties of PZT films. In studying how to control the properties of PZT by manipulation of the conditions in which it is synthesized, the lead behavior in PZT has become an important area of research [24,25,26]. The effects of formation and imperfection in PZT films on their ferroelectric polarization were explored in [27]. In [28,29], model-based representations were developed, according to which high-temperature annealing in an oxygen-containing medium of thin polycrystalline PZT films causes diffusion of lead to the grain boundary. The volatility of lead vapors during high-temperature synthesis of PZT complicates the control of the stoichiometric composition of the material [25]. The PZT exhibits a high sensitivity in its physical and its structural properties to the lead content [26,30,31]. In [25,29], it was reported that the diffusion of lead causes the formation of a lead oxide phase at the grain boundaries of the polycrystalline PZT films. It is interesting to note that, in other lead-containing materials, similar phenomena [32,33] were observed and demonstrated some commonality. The formation of a lead oxide phase may have both negative consequences in the form of deterioration of the ferroelectric properties of the PZT and positive ones in the form of new useful effects, such as the photovoltaic effect [34]. Therefore, research into the influence of lead oxide content on the kinetics of formation and the electrophysical properties of polycrystalline PZT films is very topical.

The purpose of the present work is to study the influence of lead oxide and temperature-time heat treatment regimes on the microstructure and the properties of polycrystalline PZT films in order to create a scientific basis for the controlled synthesis of ferroelectric films with defined properties.

## 2. Materials and Methods

To study the influence of lead oxide on the properties of PZT and the thin-film structures based on it, several series of samples were prepared. Thus, three series of samples of film structures were produced:

a) Capacitor structures with polycrystalline PZT films with the stoichiometric composition: Pt/PZT/Pt/Ti/SiO_2_/Si;
b) Capacitor structures of the same type as variant (a) but with PZT films with 10 mol.% of lead oxide excess;
c) Multilayer structures with PZT films and a thin sublayer of lead oxide between the ferroelectric film and the lower structure-forming electrode Pt/PZT/PbO/Pt/Ti/SiO_2_/Si.

Films of PZT and lead oxide were obtained by the method of reactive radio frequency (RF) magnetron sputtering on a specially designed piece of equipment inside the vacuum chamber of which were arranged two magnetrons with a diameter of 100 mm connected to a high-frequency generator operating at a frequency of 13.56 MHz and with a facility to vary the input power to the magnetron from 10 W to 1000 W. The target used for the sputtering of the lead oxide and the PZT films was a powder consisting of analytically pure PbO_2_ distributed in a uniform layer over the entire surface of the magnetron and industrial ferroceramic PZT with the composition Pb(Zr_0.58_Ti_0.42_)O_3_. The Zr/Ti content of PZT ceramic was selected due to the fact that its composition is near the morphotropic phase boundary and exhibits increased structural sensitivity of its properties, thus allowing the greatest possible expected changes in the material properties as a function of the lead content. As a rule, when sputtering oxygen-containing materials, a deviation in the stoichiometry of the oxygen is observed in the film obtained, and thus to compensate for the loss of oxygen in the growing films, the sputtering of the targets was performed in a gas mixture of argon and oxygen. The parameters of the deposition process are shown in Table 1.

PZT films of different thicknesses from 0.3 to 1.5 µm were formed using a two-stage method. In the first stage, the PZT films were applied by RF magnetron sputtering onto the cold substrate (≈ 130°C), allowing better control of their composition. The films were then annealed in air in a muffle furnace to induce the perovskite crystallization phase. Heat treatment was carried out using different temperature and time conditions (from 530 to 630 °C for 30–180 min). The films were heated in the furnace (ETALON, Omsk, Russia) at a rate of 10 °C/min, and the cooling was carried out at the same rate as the oven (ETALON, Omsk, Russia). To exclude various random factors, all the films were formed in a single technological deposition cycle. The lower structure-forming electrode was formed by platinum films with a thickness of 120 nm, which were deposited by the method of ion-plasma sputtering in a triple-electrode system on substrates coated with an adhesive titanium sublayer.

The magnitudes of the coercive fields and the residual polarization of the PZT films were determined from the dielectric hysteresis loops obtained by the Sawyer Tower method. The values of the dielectric constant and the tan delta angle or the dissipation factor were determined by means of an E7-12 immittance meter (Tetron, Moscow, Russia) at a frequency of 1 MHz. Investigation of the morphology of the surface layers was performed on an atomic-powered microscope (NT-MDT Spectrum Instruments, Zelenograd, Russia) with a resolution of 10 nm in the horizontal plane of the film under test and with no less than 1 nm in the vertical. The microstructure layers were investigated by SEM on a system supplied by the Helios Nanolab D449 FEI Company, Hillsboro, OR, USA. Investigations of the elemental composition were carried out on an ECO-3 Auger spectrometer (Burevestnik, St. Petersburg, Russia). The main technical parameters of the equipment were as follows: vacuum in the measuring chamber, <10^−9^ mm Hg; energy of the primary electrons, 3 keV; electron probe diameter, 5 μm; electron probe current, 5 × 10^−7^ A; resolution factor (*E*/*dE*), 250. The phase composition of the samples was studied using X-ray diffraction on a Shimadzu XRD 6000 diffractometer, Shimadzu Corp., Kyoto, Japan. The identification of the phases was carried out using the X-ray analysis database Powder Diffraction File.

## 3. Results

**Figure 1:**
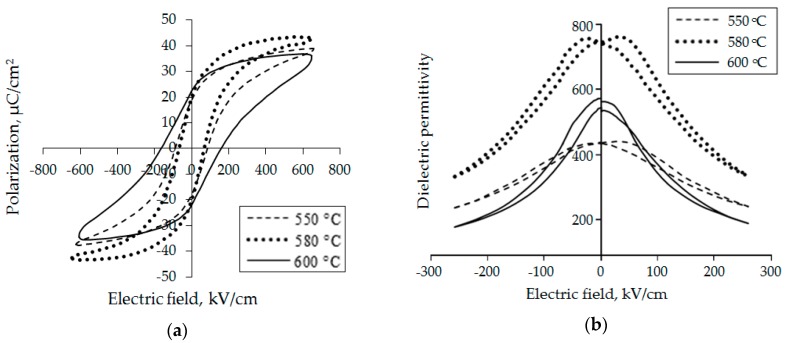
Characteristics of PZT filmsof 1.5 μm thickness formed at different crystallization temperatures: (**a**) hysteresis loops; (**b**) the dependences of the reversible dielectric permittivity on the electric field.

The measurement results of dielectric hysteresis loops and capacitance-voltage characteristics (CV) for PZT films, which were crystallized at 550 °C, 580 °C, and 600°C, are shown in Figure 1. These are the results of stoichiometric PZT films of relatively large thickness—around 1.5 μm—which was chosen in order to see the volumetric effects. The values of residual polarization (*P*_r_), coercive field (*E*_c_), control coefficient (*C*_0_/*C*_min_), and dielectric permittivity (*ε*) of PZT films were determined by experimental dependencies. The results are presented in Table 2.

These PZT films were deposited by sputtering a stoichiometric target without any lead oxide excess.

**Figure 2:**
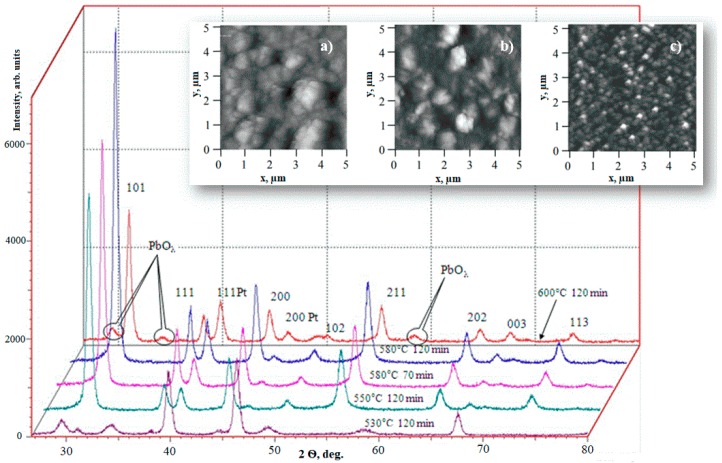
X-ray diffraction patterns of PZT films of 1.5 μm thickness formed at different temperatures and times of treatment in an oxygen-containing medium. The inset shows the surface morphology of PZT films after heat treatment in the following conditions: (**a**) 580 °C for 70 min; (**b**) 580 °C for 120 min; (**c**) 600 °C for 120 min.

The studies showed a significant effect of heat treatment parameters on the structure and the properties of PZT films. Films annealed at 550°C had low *ε*, *P*_r_, and *C*_0_/*C*_min_ values. This was due to the fact that, at this annealing temperature, the structure of the films remained heterophase (Figure 2), i.e., along with perovskite crystallites, the inclusions of pyrochlore phase were preserved. With increasing temperature, the share of pyrochlore phase decreased, which led to the growth of *ε* and *P*_r_ values at the temperature of 580 °C. At further increase of annealing temperature, the observed decrease of dielectric permeability values could be connected with formation of thin layers of lead oxide. This occurred within the intergranular space and at the interface of the thin film with platinum electrodes. The presence of the heterophase structure of PZT films was confirmed by the results of grazing incidence X-ray diffraction (GXRD). After heat treatment, PZT films had a polycrystalline structure with a preferential orientation in (110) direction, as shown in Figure 2.

With an increase of annealing temperature up to 580 °C, the degree of texturing of the films also grew. With further increase in temperature or heat treatment time, the appearance of a lead oxide phase was observed, which was accompanied by the decrease in intensity of all the reflexes from perovskite phase. It was assumed that the formation of lead oxide occurred near the interfaces and at the PZT crystallites periphery. Similarly, the structural changes associated with a decrease in the degree of ordering caused by the appearance of lead oxide should have led to a modification of the films’ surfaces.

The results of the study of the surface morphology of PZT films that were exposed to the annealing in air in different conditions showed that the film that was treated at 530 °C was distinguished by a lower surface roughness as compared to the films formed at higher temperatures. At the same time, PZT films formed at 530 °C were characterized by small dielectric constant, which indicated that the films were in the pyrochlore phase, the formation of which begins at temperatures above 450 °C.

**Figure 3:**
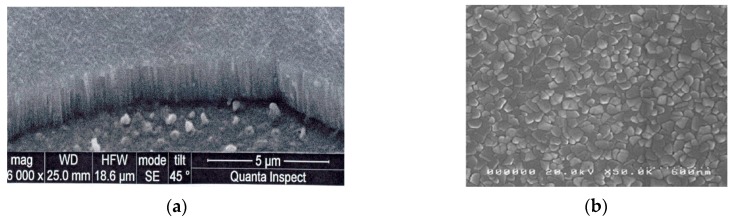
SEM images of PZT film: (**a**) side view of PZT film on Pt electrode; (**b**) PZT surface.

With an increase in the annealing temperature, the surface of the films became rougher due to the crystallization process of the perovskite phase and the formation of a columnar structure (Figure 3). The surface of PZT films formed at 580 °C for 70 min was characterized by the formation of individual 150–250 nm crystallites. Longer heat treatment at 580 °C led to a decrease in the size of the crystallite spacing and the surface roughness, and at 600 °C, they were decomposed into fully formed crystallites with a diameter of 250–350 nm. Based on the studied series of samples, the maximum dielectric constant value (770) was observed with the films annealed at 580 °C. Apparently, such low values were due to incorrect stoichiometry of the films, since the formation of PZT layers occurred without compensation for the loss of lead oxide.

The decrease in ε with increased temperature above 580°C or heat treatment times above 120 min (at 580 °C) was associated with pinning of polarization processes by charges located in the formed oxide layers of lead. It was assumed that the formation of lead oxide interlayers occurred via lead atoms located in the perovskite crystal grid. Their exit to the intergranular and the interface boundaries led to a decrease in the switching volume of the ferroelectric material, which was then accompanied by a decrease in *ε*. Also, during longer heat treatment, the thickness of PbO interlayers increased; consequently, the number of pinning charges at the interfaces also went up, which led to the removal of an even larger volume of ferroelectrics from the polarization processes. The latter was confirmed by the growth of switching fields for films annealed at 600 °C (Figure 1). The observed decrease in the switching fields and the change in the shape of the dielectric hysteresis loops with increasing heat treatment time could be associated with the formation of depolarizing fields, symmetrically located with respect to the upper and the lower electrodes.

Assuming that the dielectric constant of PbO was about 10 and the capacity of lead oxide located in the intergranular space could be neglected, the main contribution was made by PbO interface layers arranged in series with the perovskite phase with *ε* ≈ 800–900, where the total thickness of those layers was estimated at ~ 9–12 nm for films annealed at 600 °C for 120 min.

The strong pinning of polarization processes by charges in the lead oxide layers led to the presence of a rather narrow capacitive hysteresis in the CV characteristics (Figure 1b), which created conditions for the use of such films in electric field-controlled capacitors. The capacitance reproducibility for increasing and decreasing bias voltages to the capacitor structure was studied by applying a constant bias voltage from 5 to 35 V and back to 5 V for several cycles. It was found that the control ratio for PZT films annealed at 580 °C and 600 °C was 2.18, and the maximum capacity bias from the nominal value did not exceed 6%.

**Figure 4:**
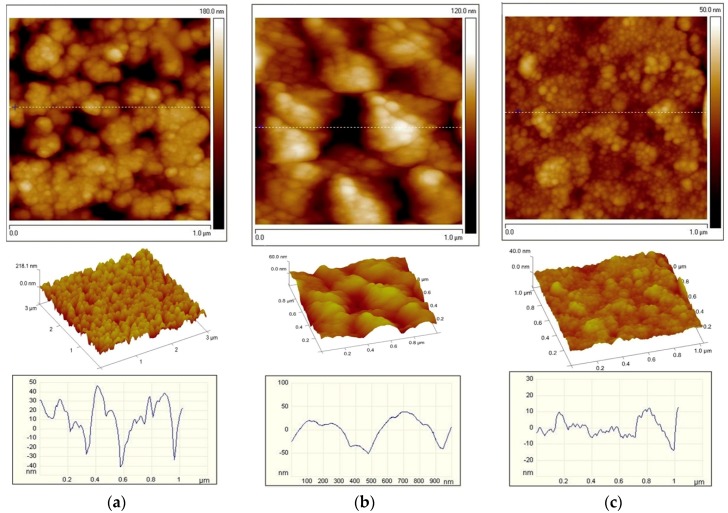
Atomic Force Microscopy (AFM) image of the PZT film surface: (**a**) without a PbO sublayer; (**b**) with 10 nm PbO thick sublayer; (**c**) with 20 nm thick PbO sublayer.

We have also studied the effect of lead oxide sublayer on PZT films and PZT-based thin-film structures properties (Figure 4). Figure 4a shows that PZT film formed at 600 °C, without a sublayer, is characterised by an average grain size of 90 nm and a surface roughness of 50 nm. A film with 10 nm sublayer has a grain size of 70 nm and roughness of 60 nm, and with 20 nm sublayer, the size of grain is 30 nm and the roughness is 10 nm. Thus, the presence of a lead oxide sublayer results in a denser formation of crystallization centers of the perovskite phase, thereby decreasing the grain size and increasing their number. This prevents the formation of grain conglomerates, which leads to a significant decrease in the surface roughness of the PZT film.

**Figure 5:**
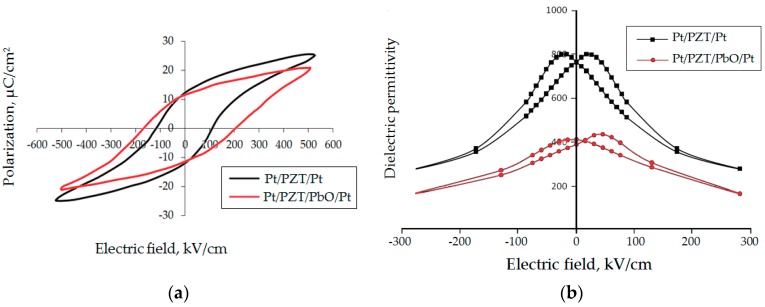
Comparison of the characteristics of PZT films with and without a PbO sublayer: (**a**) Hysteresis loops; (**b**) The dependences of the reversible dielectric permittivity on the electric field. The thickness of PZT film was 400 nm.

**Figure 6:**
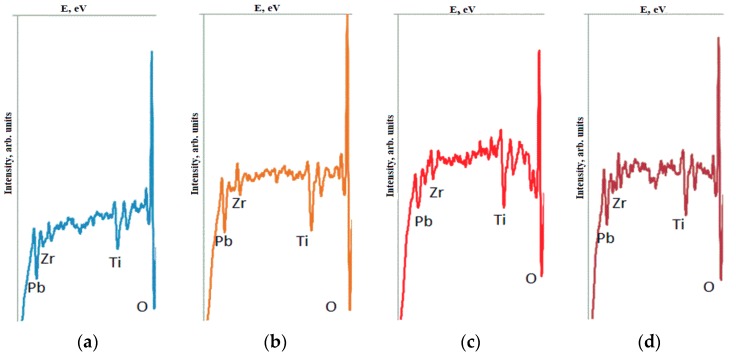
Auger spectra of PZT films: (**a**) after deposition; (**b**) after the formation of the perovskite phase; (**c**) after annealing in air; (**d**) after vacuum annealing.

The studies of the electrophysical characteristics (Figure 5) showed that the dielectric constant of the sample without a sublayer was much higher (*ε* = 760) than that of a sample with a sublayer (*ε* = 410). The CV characteristic of the sample without the sublayer was symmetrical and had a control ratio of 2.8, while the CV characteristic of the sample with the sublayer was not symmetrical and was shifted by approximately 0.7 V towards positive voltages; the control ratio was 2.4. PZT films without a sublayer and with PbO were characterized by residual polarization of 13 and 11 µC/cm^2^ and a coercive field of 110 and 185 kV/cm, respectively. It should be noted that the dielectric hysteresis loop of the PZT-PbO sample shifted to the left, which spoke to the presence of a built-in electric field of about 15 kV/cm. The results of the study showed that high-temperature heat treatment of PZT films led to the loss of lead oxide. We attempted to experimentally determine such losses with the following experiment, where 10 nm thick PZT films samples with a formed perovskite structure (the formation took place in air for 30 min at a temperature of 580 °C) were further heat treated for 180 min. The Auger spectra of PZT films samples (Figure 6) were taken immediately after RF magnetron sputtering, after the formation of the perovskite phase, and after additional annealing. Comparing the spectra of samples, the results of which are summarized in Table 3, it can be noted that, during the formation of the perovskite phase, the amount of lead oxide on the film surface decreased more intensively than during additional heat treatment. This proved that, during the formation of the perovskite structure, the diffusion of lead oxide to the film surface occurred more intensively than after its formation.

**Figure 7:**
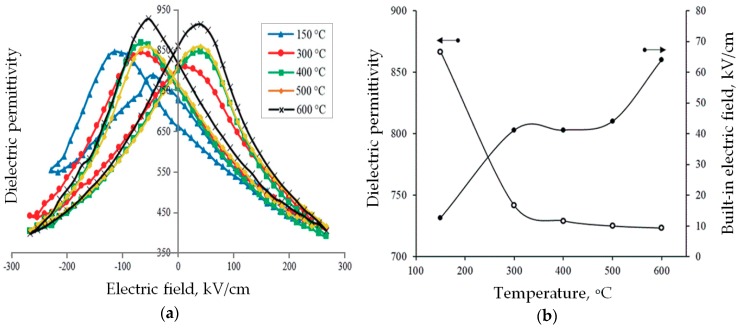
The dependences of parameters of Pt/PZT/Pt structure on the annealing temperature: (**a**) the dependence of the dielectric constant on external electric field; (**b**) the dependence of the dielectric constant and the internal electric field for the Pt/PZT/Pt structures on the annealing temperature. PZT films of 300 nm thickness were fabricated by magnetron sputtering of PZT target with 10 mol.% of PbO excess with following crystallization.

The results of the experiment agree with the concept of the formation of electrostatic charge on the free surface of the PZT film associated with the surface segregation of lead ions. When the film was cooled below the Curie temperature, this ionic charge formed a self-polarized PZT state (Figure 7).

After deposition of the upper platinum electrodes (at ~150°C), the capacitance-voltage characteristics of the structure had a maximum internal field. With an increase in the annealing temperature to the Curie value (300 °C), the effect of self-polarization decreased, since the conditions at the upper boundary of the PZT film had already changed (the metal shields the surface ionic charge), and the domain structure of the film after cooling formed in a much smaller field of the pre-surface layer. With an increase in the annealing temperature up to 600 °C, the capacitance increased, and the internal field practically disappeared due to the diffusive equalization of the PZT composition in the pre-electrode layer and its partial transition to the perovskite phase.

## 4. Discussion

**Figure 8:**
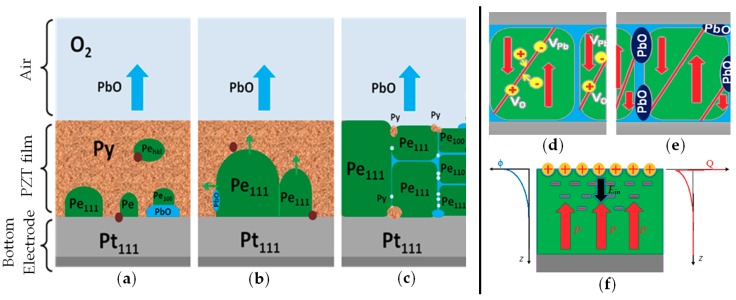
The processes during the formation of PZT films at the annealing and the effect of local stoichiometry disturbances on the polarization pinning in the resulting PZT films: (**a**) nucleation of the perovskite (Pe) phase in a pyrochlore (Py) or amorphous matrix on various inhomogeneities; (**b**) growth of perovskite crystallites; (**c**) grain boundary segregation of PbO_λ_; (**d**) domain wall pinning in PZT grains by ionized vacancies; (**e**) PbO_λ_ inclusions and polarization pinning; (**f**) self-polarized film due to the double charge defect layer.

The variety of processes during the formation of PZT films at the annealing stage are depicted in Figure 8a–c. Figure 8a shows nucleation of the perovskite (Pe) phase in a pyrochlore (Py) or amorphous matrix (depending on the film deposition conditions) on various inhomogeneities (substrate, defects, PbO_λ_ inclusions).Figure 8b shows growth of perovskite crystallites in the pyrochlore matrix limited by segregation of excess PbO_λ_, by other defects, and by the growth of neighboring grains. Figure 8c shows the final stage of crystallization of the film, the grain boundary segregation of PbO_λ_, and the formation of intergranular precipitates of PbO_λ_. The formed film was an open system and exchanged oxygen with the vapor-gas medium; in addition, partial removal of the most volatile component, PbO_λ_ molecules, from the film was possible. Figure 8 also illustrates the effect of local stoichiometry disturbances on the polarization pinning in the resulting PZT films.

The study showed a significant effect of heat treatment parameters on the structure and the properties of PZT films. It was shown (Figure 2) that, even in the original stoichiometric PZT film, under certain conditions (temperature above 580 °C, duration greater than 70 min), impurities of lead oxide could be formed. This was because the thermal treatment led to partial decomposition of the PZT grain with the removal of the most active oxide (i.e., lead oxide) based on the following reaction:

{Pb(Zr, Ti)O_3_}*_N_*→Pb*_N_*_−*m*_(Zr, Ti)*_N_*O_3*N*−*m*λ_ + *m*PbO_λ_ + *m*V_Pb_ + *m*λV_O_ (1)

where PbO_λ_ is a lead oxide molecule that enters the PZT grain boundary; *N* is the total number of particles in the grain; *m* is the number of particles removed from the grain; V_Pb_ and V_O_ are lead and oxygen vacancies (at crystallization temperatures of PZT, they are ionized). The presence of an increased number of ionized vacancies in PZT grains could lead to domain wall pinning (Figure 8d), which could result in a decreased volume of the ferroelectric film switched by the field. This affected the decrease of the nonlinearity in the CV characteristic and the decrease of the rectangularity of hysteresis loops, as shown in Figure 1. Equation (1) could be reversed if the formed material had an excess of lead. However, an increase in lead oxide inclusions also led to a decrease in the effective switching volume of the PZT grains (Figure 8e), also due to the increased conductivity of lead oxide.

The choice of the optimal value of the lead concentration was not so trivial and was associated with the tasks being solved. The diffusion of lead ions in the film led to the formation of a positive charge on the free surface of the PZT film (Figure 8f). The release of lead ions to the surface was associated with the formation of negatively charged vacancies in the bulk of PZT. Thus, a double charged layer was formed. Oxygen ions and oxygen vacancies partially compensated for these charges, but not completely, since the formation energies of oxygen and lead vacancies in PZT were very different. The enthalpy of formation of an ionized vacancy in oxygen was 5.4 eV and in lead was 3.3 eV.

Let us try to estimate the surface potential associated with the segregation of lead ions. Consider the case of a quasi-chemical equilibrium “PZT film surface-gas” (at a crystallization temperature of PZT) in which the atoms in the film are mobile and where both electrons and atoms can be rearranged. This leads to a change in the number of all defects, particularly to the release of Pb^2+^ and O^2−^ ions to the PZT film surface. The following also occur: surface charge compensated by the space charge in the volume of the film causes the appearance of an electric potential, the concentration of individual defects varies according to the Boltzmann ratio, and the space charge density and the field potential are related by the Poisson equation. In this case, it is necessary to take into account the equilibrium of atomic and electronic defects. Thus, for a PZT film, in the case of Schottky volume disordering, we may derive as follows: $\left[V_{\mathrm{Pb}}^{\prime \prime }\right]=\left[V_{O}^{••}\right]=K_{S}^{1/2}$. Hereinafter in the formulas Kreger’s notation is used [35].

The surface charge arises due to the fact that the energy of the formation of vacancies by removing ions from the crystal to the surface varies for both types of ions. The concentrations of Pb^2+^ and O^2−^ ions on the surface of the crystal are caused by the difference in the vacancy concentrations inside PZT. The formulation of the equations of reactions, according to which individual vacancies are formed, is complicated by the fact that the idea of the effective charge and vacancies, to some extent, loses its meaning near the surface. In this regard, we use these concepts formally to explain surface phenomena.

Let’s assume that in certain places of the surface there are Pb^2+^ ions and in others, O^2−^ ions. Denote the first positions by S_Pb,S_, the second by S_O,S_. As a rule, these are not any places on the surface, but places on the edges and corners of the growing face (i.e., so-called places of self-generation).

It should be noted that, likewise inside PZT, S_Pb,S_ are vacancies near oxygen ions O^2−^ and S_O,S_ are vacancies near lead ions Pb^2+^. As a result, the capture by the O^2−^ ion of the unoccupied space of S_O,S_ contributes to the formation of a new unoccupied space S_Pb,S_ and vice versa. Therefore, the transfer of Pb^2+^ and O^2−^ from the PZT volume to the surface can be put down as follows:

(2) $${\mathrm{Pb}}_{\mathrm{Pb}}^{x}+S_{\mathrm{Pb},S}^{x}\to {\mathrm{Pb}}_{\mathrm{Pb},S}^{2+}+V_{\mathrm{Pb}}^{\prime \prime }+S_{O,S}^{x};\;O_{O}^{x}+S_{O,S}^{x}\to O_{O,S}^{2-}+V_{O}^{••}+S_{\mathrm{Pb},S}^{x}$$

The electrochemical potentials η*_i_* for structural elements with effective chemical potentials ξ*_i_* and *z_i_q* charges are determined by the equation η*_i_* = ξ*_i_ + z_i_q*ϕ, where ϕ is the electric potential. For any reaction in which structural elements with ν*_i_* number take part, at equilibrium: $\underset{i}{\sum }v_{i}\eta_{i}=0$. From (2):

(3) $$\eta \left(V_{\mathrm{Pb}}^{\prime \prime }\right)+\eta \left({\mathrm{Pb}}_{\mathrm{Pb},S}^{2+}\right)-\eta \left({\mathrm{Pb}}_{\mathrm{Pb}}^{x}\right)+\eta \left(S_{O,S}^{x}\right)-\eta \left(S_{\mathrm{Pb},S}^{x}\right)=0$$

Equation (3) looks the same for oxygen ions. For uncharged structural elements η*_i_* = ξ*_i_*. If you accept that inside the PZT film (*x* = ∞) ϕ = 0, and on the surface (*x* = 0) ϕ = ϕ_0_, then inside the film: $\eta \left(V_{\mathrm{Pb}}^{\prime \prime }\right)=\xi \left(V_{\mathrm{Pb}}^{\prime \prime }\right)$. Given that $\xi \left({\mathrm{Pb}}_{\mathrm{Pb},S}^{2+}\right)\approx \xi \left({\mathrm{Pb}}_{\mathrm{Pb}}^{x}\right)$, the Equation (3) is simplified to (and the same is for oxygen ions):

(4) $$\xi {\left(V_{\mathrm{Pb}}^{\prime \prime }\right)}_{\infty }+\xi \left(S_{O,S}^{x}\right)-\xi \left({\mathrm{Pb}}_{\mathrm{Pb},S}^{x}\right)=-2q\phi_{0};\;\xi {\left(V_{O}^{••}\right)}_{\infty }-\xi \left(S_{O,S}^{x}\right)+\xi \left({\mathrm{Pb}}_{\mathrm{Pb},S}^{x}\right)=2q\phi_{0}$$

The dependence of the potential on the concentration can be represented as: $\xi_{i}=\xi_{i}^{0}+kT\ln\left[i\right]$, where [*i*] corresponds to the proportion of places occupied by the structural element *i*; *k* and *T* are Boltzmann constant and temperature. Since *ξ^0^* values are independent of ϕ, they are also independent of *x*. Then, we may derive as follows:

(5) $$4q\phi_{0}=\;G\left(V_{O}^{••}\right)-G\left(V_{\mathrm{Pb}}^{\prime \prime }\right)+kT\left\{\ln\frac{{\left[V_{O}^{••}\right]}_{\infty }}{{\left[V_{\mathrm{Pb}}^{\prime \prime }\right]}_{\infty }}+2\ln\frac{\left[S_{O,S}^{x}\right]}{\left[S_{\mathrm{Pb},S}^{x}\right]}\right\}\approx H\left(V_{O}^{••}\right)-H\left(V_{\mathrm{Pb}}^{\prime \prime }\right)\approx 2.1\;\mathrm{eV}$$

where *G* and *H* are the Gibbs energy and enthalpy of formation, respectively.

Since the electrochemical potential of each particle is constant throughout the system,

(6) $$\eta_{i}=\xi_{i}^{0}+kT\ln\left[i\right]+zq_{i}\phi =\xi_{i}^{0}+kT\ln{\left[i\right]}_{\infty }$$

Hence: $kT\ln\left[i\right]/{\left[i\right]}_{\infty }=-zq_{i}\phi$ is applicable to lead and oxygen vacancies, then for the potential and the space charge distribution we get as follows:

(7) $$\frac{\partial^{2}\phi }{{\partial_{x}}^{2}}=\frac{2q}{\varepsilon }\left\{\left[V_{\mathrm{Pb}}^{\prime \prime }\right]-\left[V_{O}^{••}\right]\right\}=\frac{2q}{\varepsilon }\left\{{\left[V_{\mathrm{Pb}}^{\prime \prime }\right]}_{\infty }\exp\left(-2\phi q/kT\right)-{\left[V_{O}^{••}\right]}_{\infty }\exp\left(2\phi q/kT\right)\right\}$$

The estimates show that, with small stoichiometry bias, the electrostatic potential associated with the segregation of lead ions onto the free surface of PZT films can reach 0.5 V. This potential is quite enough to cause a self-polarization state of PZT film when it is cooled below Curie temperature (due to the small coercive field). Further, when the film is cooled to room temperature, the self-polarized state becomes stabilized and the migration of lead ions occurs. The destruction of the self-polarized state is possible by repeated thermal treatment of the film above the Curie temperature with the deposited upper electrode, as shown in Figure 7.

The formation of double charged layers in PZT films is possible not only at the PZT-air interface but also at the PZT grain boundaries in a polycrystalline film. The mechanisms of such formations are similar.

We did not see any significant shifts in the hysteresis loops or the capacitance-voltage characteristics in Figure 1, since these were the results of stoichiometric PZT films of relatively large thickness (around 1.5 μm). Such a thickness was chosen in order to see the volumetric effect of lead output on the grain periphery (Figure 2) according to Equation (1) and to minimize the surface effect at the film–air interface. Considering that the grain size of the PZT was about 90 nm (Figure 4), about ten grains of the PZT fit into the film thickness. Therefore, when lead ions went to PZT grain surfaces, the internal fields were oriented in different directions. The result of this was the absence of a shift in characteristics but the appearance of a narrow capacitance-voltage hysteresis.

Figure 7, on the contrary, shows the built-in field because the thickness of the PZT film was about 300 nm and contained 10 mol.% excess of lead. We observed the effect associated with the release of lead on the free surface (upper interface of the film). This led to the formation of a built-in field at the upper interface and appeared in a strong left shift of the capacitance-voltage characteristic (and we saw a similar shift on hysteresis loops).

When a lead oxide sublayer was added at the bottom interface of the PZT film, we also observed a shift in the characteristics (Figure 5), but this shift was already in the other direction.

These effects could be positive and practically interesting for obtaining stable self-polarized films without applying an external field (in order to use in pyroelectric based sensors, microelectromechanical devices, and so on). Another application is the production of non-linear capacitors with very narrow CV hysteresis. The future development of self-polarized PZT films will be associated with the production of multilayer structures and the study of their properties. In particular, developments in the field of creating ferroelectric superlattices [21,22,23] where PZT can be used look very promising.

## 5. Conclusions

This work showed that increasing the temperature or the duration of heat treatment of PZT films could lead to the appearance of impurities of lead oxide, even if the PZT film did not originally contain a super-stoichiometric excess of lead, which was a non-obvious finding. It was also shown that, even in the original stoichiometric PZT film under certain conditions (temperature above 580 °C, duration greater than 70 min), impurities of lead oxide could be formed.

It was shown that the presence of a sublayer of lead oxide led to a denser formation of crystallization centers of the perovskite phase, resulting in a reduction of the grain size and the emergence of a charge on the lower interface, which gave rise to the presence of an embedded field, leading to the self-polarization of the PZT film.

Pinning of the polarization processes by charges, which were formed at the stage of the formation of the film due to the diffusion of lead ions, caused a decrease in the values of the dielectric constant and the growth of transition fields and the presence of a narrow capacitance hysteresis (or lack of it) on the capacity-voltage characteristic.

The formation of the perovskite structure under high-temperature annealing was accompanied by the diffusion of lead into the surface of the film. It was found that the diffusion of lead oxide to the surface of the film occurred more intensely during the formation of the perovskite structure than after its formation.

The temperature and the time regimes of the formation of Pt/PZT/Pt structures influenced the formation of the self-polarized PZT film, which could be attributed to the accumulation and the relaxation of an electrostatic charge on the free surface of the ferroelectric film associated with surface segregation of the lead ions.

## Figures and Tables

**Table 1:** Parameters of the deposition processes for PbO and lead zirconate-titanate (PZT) films.

Parameters of Technological Process	PbO	PZT
Power of radio frequency (RF) discharge, watt	38	100
Diameter of target, mm	100	100
Composition of gaseous mixture	76%Ar + 24%O_2_	76%Ar + 24%O_2_
Working pressure of gaseous mixture, mm Hg	10^−2^	10^−2^
Temperature of substrate, °C	130 or 400	130
Growth rate of films, nm/min	10	5.8

**Table 2:** Electro-physical parameters of PZT films.

Heat Treatment Conditions		*ε*	tgδ	*P*_r_,µC/cm^2^	*E*_c_,kV/cm	*C*_0_/*C*_min_
*T*, °C	*t*, min					
530	70	120	0.085	-	-	1
530	120	70	0.019	-	-	1
550	70	400	0.176	18	87	1.9
550	120	350	0.22	14	67	1.8
580	70	770	0.165	20	68	2.5
580	120	730	0.174	14.5	42	2.6
600	70	750	0.09	22	162	3.1
600	120	570	0.085	17	152	2.6
630	70	560	0.12	19	171	2.5
630	120	510	0.11	14	160	2.3

**Table 3:** Intensities of PZT films components signal determined on the basis of Auger spectra.

PZT Films	Pb	O	Ti	Zr
After deposition on the “cold” substrate	3	9.75	2.25	1
After the formation of the perovskite phase	2.5	10.5	3	1
After repeated annealing in air	1.5	8	3.25	1
After vacuum annealing	1.5	7	3	1
